# Quenching of tryptophan fluorescence in a highly scattering solution: Insights on protein localization in a lung surfactant formulation

**DOI:** 10.1371/journal.pone.0201926

**Published:** 2018-08-03

**Authors:** Luca Ronda, Barbara Pioselli, Silvia Catinella, Fabrizio Salomone, Marialaura Marchetti, Stefano Bettati

**Affiliations:** 1 Department of Medicine and Surgery, University of Parma, Parma, Italy; 2 Biopharmanet-TEC, University of Parma, Parma, Italy; 3 Chiesi Farmaceutici, R & D Department, Parma, Italy; 4 Italian National Institute of Biostructures and Biosystems, Rome, Italy; Rijksuniversiteit Groningen, NETHERLANDS

## Abstract

CHF5633 (Chiesi Farmaceutici, Italy) is a synthetic surfactant developed for respiratory distress syndrome replacement therapy in pre-term newborn infants. CHF5633 contains two phospholipids (dipalmitoylphosphatidylcholine and 1-palmitoyl-2oleoyl-*sn*-glycero-3-phosphoglycerol sodium salt), and peptide analogues of surfactant protein C (SP-C analogue) and surfactant protein B (SP-B analogue). Both proteins are fundamental for an optimal surfactant activity in vivo and SP-B genetic deficiency causes lethal respiratory failure after birth. Fluorescence emission of the only tryptophan residue present in SP-B analogue (SP-C analogue has none) could in principle be exploited to probe SP-B analogue conformation, localization and interaction with other components of the pharmaceutical formulation. However, the high light scattering activity of the multi-lamellar vesicles suspension characterizing the pharmaceutical surfactant formulation represents a challenge for such studies. We show here that quenching of tryptophan fluorescence and Singular Value Decomposition analysis can be used to accurately calculate and subtract background scattering. The results indicate, with respect to Trp microenvironment, a conformationally homogeneous population of SP-B. Trp is highly accessible to the water phase, suggesting a surficial localization on the membrane of phospholipid vesicles, similarly to what observed for full length SP-B in natural lung surfactant, and supporting an analogous role in protein anchoring to the lipid phase.

## Introduction

Mammalian lung surfactants are mixtures of 90% lipids and about 10% of four surfactant-specific proteins named SP-A, SP-B, SP-C, and SP-D [1] with the role of reducing surface tension in the alveolar space, thus allowing breathing, uniform lung inflation and preventing alveolar collapse during expiration [2, 3]. Pre-term newborns, lacking functional pulmonary surfactant, experience respiratory distress syndrome (RDS). This condition is clinically treated with surfactant replacement therapy. Therapeutic pulmonary surfactants preparations are mixtures of lipids and lipids-interacting proteins with the role of sustaining lung surfactant function [2, 3].

SP-A and SP-D proteins are large hydrophilic macromolecules belonging to the family of collectins [3]. These two components are collagenous glycoproteins mainly involved in antibody-independent pulmonary host defence [4, 5]. While SP-A is usually associated with pulmonary surfactant membranes, SP-D is not commonly obtained from alveolar lavage and, then, is not considered a membrane-associated surfactant protein. Both molecules have also been proved to influence surfactant homeostasis in addition to their role in innate host defence [4]. SP-B and SP-C are the hydrophobic proteins mainly responsible for the adsorption and spreading of the surfactant film at the air-liquid interface. Both surfactant proteins SP-B and SP-C (or their analogues) were demonstrated to be necessary for an optimal surfactant activity as they improve the reduction of surface tension [6, 7] and avoid alveoli collapsing at the end of expiration in a rabbit model of RDS [8, 9]. As a matter of fact, humans and animals with deficiency in SP-C develop severe respiratory diseases, while SP-B genetic deficiency in humans and mice is a cause of lethal respiratory failure after birth [10, 11]. SP-B is the most relevant protein for surfactant physiology. It has a complex structure belonging to the saposin-like proteins family and it is known to permanently interact with membranes through 4- to 5-amphipathic helical bundles [3]. The structural arrangement of the disulfide bridges in SP-B is highly conserved in invariant positions and involves two intra-molecular disulfide bonds and one inter-molecular disulfide bond which characterizes the covalent homodimers of mature SP-B obtained from alveolar space [3, 12, 13]. Due to its high hydrophobicity SP-B is poorly water-soluble while it is soluble in organic solvents like chloroform and methanol. Some structural insights on the mode of interaction of SP-B with labelled lipids have emerged from Forster resonance energy transfer (FRET) measurements [14]. SP-B homodimer seems to induce lipid restructuring in particular by interaction with anionic lipids. This appears to be a key aspect of the function of SP-B, characterized by a strongly amphipathic structure, with most of the positively charged aminoacid side chains on one face and a hydrophobic patch on the other face [15]. This picture is consistent with a topology model where SP-B occupies a superficial position with respect to membranes (19), with no deep penetration into the surfactant bilayers, catalyzing the movement of phospholipids from surfactant membranes into the active air-liquid interface [3]. Particularly, a relevant structure-activity feature of SP-B with respect to the interaction with lipids at the interface is represented by the presence of aromatic aminoacids at the N-terminus [16].

On the other hand, SP-C is a hydrophobic protein, formed by an alpha helix with an esterified palmitoyl group at the N-terminal [17]. The helical segment is fully inserted in the lipid bilayer parallel to the acyl groups [18, 19]. It has been reported that SP-C, as well as SP-B, is spread into disordered fluid-like membrane phases [20] where its role is to store up the unsaturated lipid components increasing the collapse pressure of the surfactant film.

Different attempts to develop a synthetic surfactant have been made, with a special effort towards design and synthesis of SP-B and SP-C analogues [21–25]. The main obstacles to be overcome in the design and synthesis of SP-B and SP-C peptide analogues are the relatively complex tertiary conformation of SP-B and the metastable structure of SP-C.

CHF5633 is a fully synthetic surfactant synthesized by Chiesi Farmaceutici (Italy) designed to treat RDS in pre-term newborn infants as a surfactant replacement therapy. CHF5633 contains dipalmitoylphosphatidylcholine (DPPC), 1-palmitoyl-2oleoyl-*sn*-glycero-3-phosphoglycerol sodium salt (POPG), a surfactant protein C analogue (SP-C analogue) and a surfactant protein B analogue (SP-B analogue) [26–28].

Given the high sensitivity of fluorescence as a standard technique to investigate protein conformation and the local environment of chromophoric groups [29, 30], we decided to exploit the fluorescence emission of the single tryptophan residue of the SP-B analogue (SP-C analogue has none) to characterize local microenvironment and protein interaction with phospholipids within the synthetic surfactant. Trp fluorescence has already been exploited as a probe to investigate the localization and the mode of interaction with lipid layers of full length SP-B and peptide analogues in aqueous solvents or dilute model membranes or micelles. In fact, Trp9 in SP-B is found to be critical for optimal interface affinity [15, 31]. In the SP-B analogue contained in the CH5633 formulation, comprising residues 8–23 and 63–78 of the full-length protein, Trp is in position 2, corresponding to Trp9 of the full length peptide, and seems not to take part to the interhelix interaction, but to the anchoring of the peptide at the lipid-water interface. Similar to Trp9 in SP-B, its location in detergent micelles is in between the hydrophobic face and the positively charged one and extends out from the protein surface in a position suitable to anchor SP-B analogue to the lipid phase [15].

In order to exploit Trp fluorescence to gain insight on SP-B analogue structure and mode of interaction with multilamellar vesicles in the CHF5633 synthetic surfactant as it is, without any extraction process or dilution, we had to overcome the high light scattering activity of the suspension, due to the multi-lamellar vesicles structure of the lipid components, leading to very bad quality fluorescence emission spectra. We envisaged a strategy consisting in tryptophan fluorescence quenching by acrylamide at increasing concentrations, followed by back-extrapolation of background scattering through a Singular Value Decomposition (SVD) analysis procedure. Fluorescence quenching is, indeed, a powerful method for studying the solvent accessibility of chromophoric groups [32, 33] and the localization of fluorescent molecules within macromolecular assemblies such as lipid bilayers. In this regard, several studies have been carried out on peptides and proteins bound or inserted into a lipid bilayer, and fluorescence quenching allowed for instance to determine the depth of penetration of tryptophan residues into membranes, contributing to the generation of models for the interaction of several proteins with membranes [34, 35]. In the present study, we report a characterization of the CHF5633 synthetic surfactant, with special regard to local structural homogeneity of the SP-B analogue, the solvent exposure of its single tryptophan residue, and peptide interaction with phospholipids. The results of this study, the first one to our knowledge to exploit fluorescence spectroscopy on an intact pharmaceutical formulation of lung surfactant, are consistent with previous studies on SP-B analogue in micelles and model membranes [15] and provide new insights on its topology and mode of interaction with lipid structures within the synthetic surfactant CHF5633. The procedure we followed to correct Trp fluorescence emission spectra from the dominant contribution of scattering could in principle be extended to other biological fluids or pharmaceutical preparations characterized by strong light diffusing properties.

## Materials and methods

### Reagents

All reagents were of the best commercially available quality and were used without further purification.

CHF5633 synthetic surfactant formulation, provided by Chiesi Farmaceutici (Parma, Italy) is composed by 1:1 dipalmitoylphosphatidylcholine (DPPC) (39.32 mg/ml), 1-palmitoyl-2oleoyl-*sn*-glycero-3-phosphoglycerol sodium salt (POPG) (39.32 mg/ml), SP-B analogue (CWLCRALIKRIQALIPKGGRLLPQLVCRLVLRCS) (0.2% w/v) and SP-C analogue (**IPSSPVHLKRLKLLLLLLLLILLLILGALLLGL**) (1.5% w/v). The final product is a sterile suspension with a total concentration (phospholipids + peptides) of 80 mg/ml.

Acrylamide solution (40% w/v) was purchased from Biorad (Milan, Italy). Methanol was from Sigma-Aldrich (St. Louis, USA).

### Fluorescence instrumentation and measurements

Fluorescence spectra were recorded with a Horiba Fluoromax 3 (Jobin Yvon, Kyoto, Japan) spectrofluorimeter equipped with a thermostatted bath. Samples were contained in a 5 x 5 mm quartz cuvette, maintained at 20 ± 0.5°C. The slits width was set to 2.5 nm and the integration time to 0.4 seconds to optimize the signal to noise ratio.

For tryptophan fluorescence quenching by acrylamide, 700 μL of CHF5633 formulation are gently poured into the cuvette and the emission spectrum (unquenched tryptophan) is recorded. Upon every acrylamide addition, the solution containing the quencher is mixed with the formulation by manual inversion, avoiding vigorous shaking to minimize bubbles formation. The sample is then left to equilibrate at 20°C in a thermostatted cell holder for 5 minutes, then the cuvette is rapidly extracted, mixed again by manual inversion (6–7 fold) and repositioned in the cell holder. Measurements are started after 60–70 seconds. This last mixing procedure is repeated before each spectrum collection. At each acrylamide concentration, 3 spectra were collected.

The fluorescence quenching experiments with 10-doxyl nonadecane (10-DN) were carried out following the same procedure as those performed with acrylamide, except for the fact that: i) the quencher, which is dissolved in ethanol, is deposited on the cap of the cuvette, and ethanol is left to evaporate (dry sample preparation method) [36] before mixing by manual inversion of the cuvette; ii) further mixing by 2 minutes of manual inversion, followed by 10 minutes of gentle stirring (30–40 rpm), was carried out before thermostatting the cuvette.

### Data analysis

Singular value decomposition (SVD) [37, 38] and data fitting were carried out using MATLAB R2015b.

In SVD analysis, a given (m x n) data matrix (A), where m is the number of wavelengths and n is the number of collected spectra, is resolved into a product of three matrices, usually named U, S, and V^T^, as follows:

$$A=U\;X\;S\;X\;V^{T}$$

U matrix consists of n orthonormal eigenvectors, S is a square diagonal matrix, and V^T^ is a matrix whose rows are also orthonormal. The columns of U are the spectral components. For each U component, the acrylamide concentration dependence is derived from the corresponding row in the V^T^ matrix. The main criterion for the selection of usable components is the magnitude of the singular values, the higher values being the meaningful ones.

Graphical representation of data was prepared using the software SigmaPlot 12.0.

## Results and discussions

### Tryptophan fluorescence emission of SP-B analogue in synthetic surfactant formulation: A mechanical explanation for the time evolution of signal intensity

**Fig 1:**
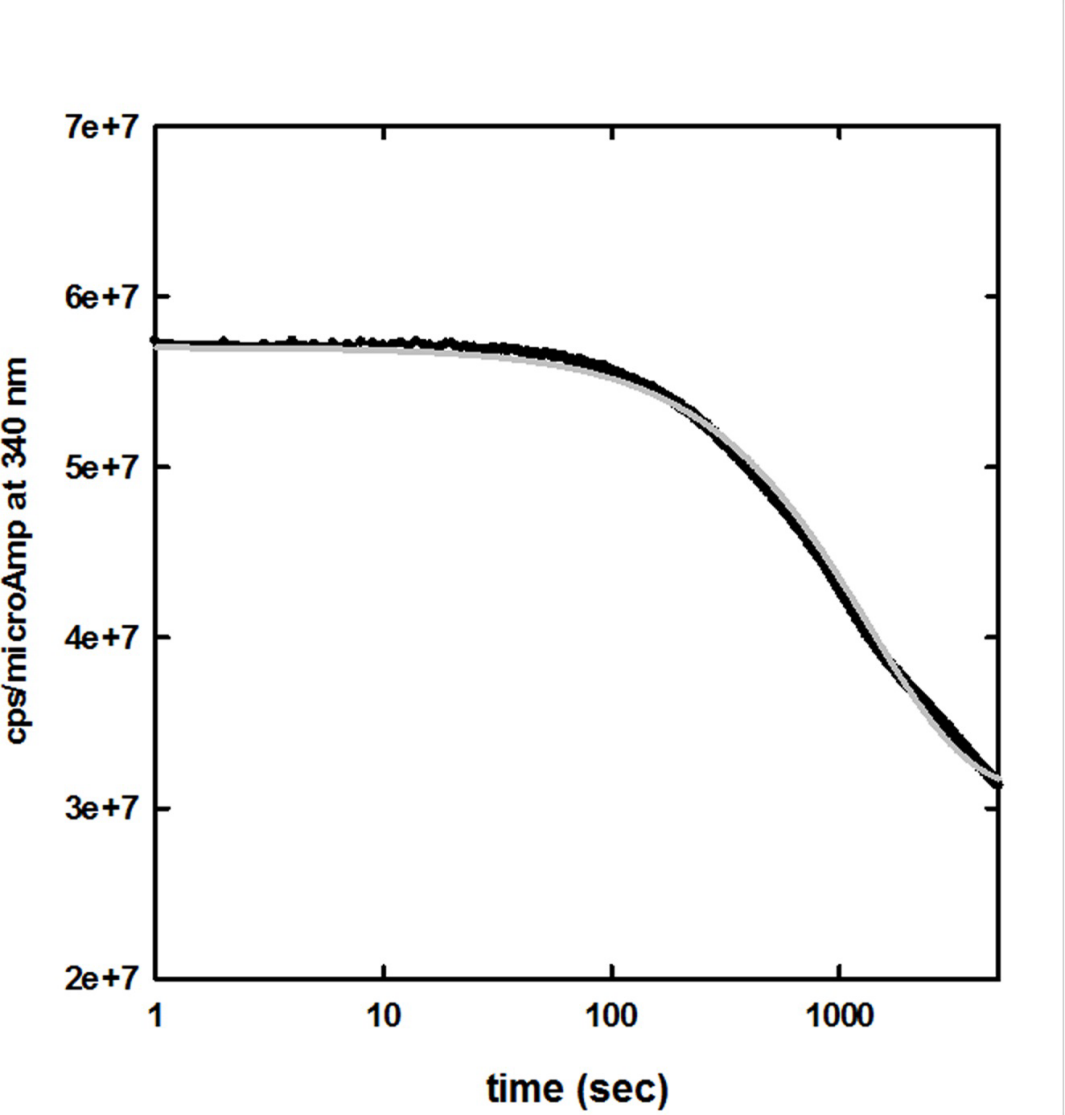
Fluorescence emission at 340 nm of CHF5633 synthetic surfactant upon excitation at 280 nm, as a function of time, at 20 ± 0.5°C. The observed time evolution (black line, average of three different kinetic traces) was fitted to a stretched exponential function (grey line), yielding a relaxation time of 1000 sec and a β stretching factor of 0.77.

We first evaluated the intensity and the stability of Trp fluorescence emission in the CHF5633 synthetic surfactant formulation by monitoring the fluorescence emission at 340 nm upon excitation at 280 nm (Fig 1). At this excitation wavelength, only the single Trp residue of SP-B analogue is expected to contribute significantly to fluorescence emission by the synthetic surfactant, since no Trp is present in the sequence of SP-C analogue. Acquisition of fluorescence emission signal was started immediately after mixing of the sample by manual inversion, and extended to 90 min.

The time evolution of fluorescence emission is well described by a stretched exponential function, in which a fractional exponent (β) is present in the exponential function:

$$f\left(t\right)=y_{o}+a\cdot e^{{\left(-\frac{t}{\tau }\right)}^{\beta }}$$

The fit yielded a relaxation time τ around 1000 sec (Fig 1) and a stretching factor β of 0.77. This experiment indicates that the time required for acquisition of a whole emission spectrum under the present experimental settings (about 60 seconds) is short enough to avoid any significant drift in the fluorescence emission. On the other side, the slow decay in signal intensity, which is reversible upon mixing of the sample (data not shown), is perfectly consistent with the hypothesis that within the synthetic surfactant the predominant form of SP-B analogue is not that of freely diffusing monomers or oligomers, but rather it is stably adherent to the multilamellar lipid vesicles of the formulation. In fact, if we consider the fluorescent decay as due to the probe sedimentation in the cuvette, the sedimentation rate will be: $v=\frac{2}{9}\cdot \frac{r^{2}\cdot g\cdot \left(\rho_{p}-\rho_{fluid}\right)}{\eta }$ where r is the radius, ρ_p_ (about 1.02 g/cm^3^) the density of the particles, and ρ_fluid_ and η are the density and the viscosity (10 cPoise) of the fluid. Assuming multilamellar vesicles with a broad radius distribution centered at around 10 μm range [39], the expected sedimentation rate is of the order of 10^−6^ m/s. As for the stretched exponential model, a huge number of relaxation phenomena, in particular complex systems, have been shown to be well described by this function. A lower stretching factor indicates a higher heterogeneity of the system. A stretching factor of 0.77 is consistent with the current assumptions and results, with a broad distribution of vesicles size, implying a distribution of sedimentation rates. A detailed characterization of this particular aspect, that could be pursued e.g. with light scattering techniques, is beyond the scope of the present work.

### Tryptophan fluorescence emission of SP-B analogue in synthetic surfactant formulation: Expedient baseline generation by quenching experiments

Due to the high sensitivity of fluorescence to the microenvironment of chromophoric groups [29, 30] we intended to take advantage of the fluorescence emission of the single tryptophan residue of SP-B analogue to investigate the environment of the peptide in the synthetic surfactant. Moreover, we planned to exploit fluorescence quenching [32, 33] to gain further insight into Trp intramolecular localization, and SP-B analogue positioning within the highly structured synthetic surfactant matrix.

**Fig 2:**
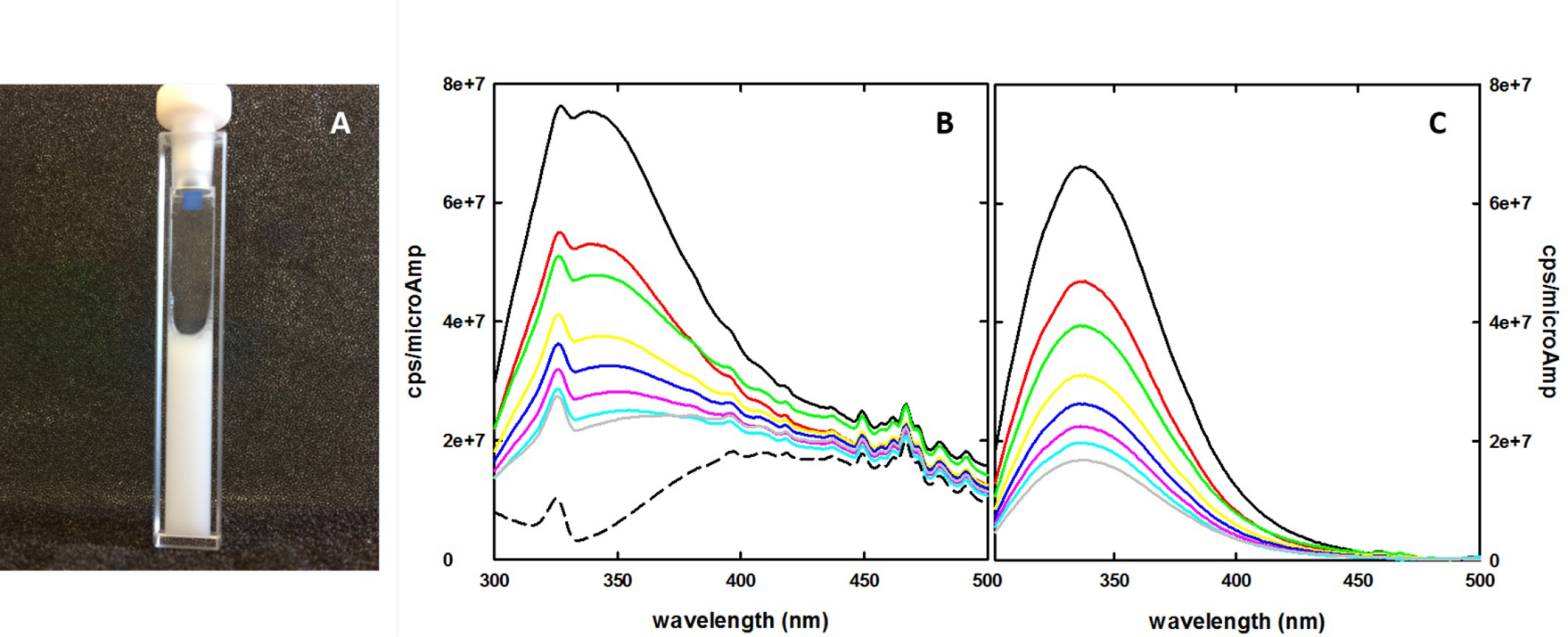
A: CHF5633 synthetic surfactant in a quartz cuvette used for fluorescence measurements. The formulation presents as a thick, white suspension. B: Fluorescence emission spectra of CHF5633 synthetic surfactant in the absence and presence of acrylamide, upon excitation at 280 nm, at 20 ± 0.5°C, in the absence (continuous black line) and presence of different concentrations of acrylamide (50 mM, red, 100 mM, green, 150 mM, yellow, 200 mM, blue, 250 mM, magenta, 300 mM, light blue, 400 mM, grey). The dashed black line indicates the extrapolated baseline, calculated following the procedure reported in the text. C: Emission spectra from panel B, after correction for the calculated baseline.

Unfortunately, the CHF5633 synthetic surfactant formulation presents in the form of a white uniform suspension (Fig 2A), this making fluorescence measurement challenging, particularly in the UV range. Once proper instrumental parameters were defined in order to yield a good signal intensity in single wavelength experiments using a standard 5 x 5 mm cuvette (see above), reproducible steady-state emission spectra were collected. However, while an acceptable signal to noise ratio was obtained with a right angle configuration, there remained the critical issue of the lack of a proper baseline for background correction of emission spectra (Fig 2B).

In fact, the fluorescence emission spectra were disturbed by a large scattering effect, overwhelming "true" fluorescence emission. Moreover, CHF5633 synthetic surfactant is a formulation that intrinsically contains the fluorescent probe, i.e. the Trp of SP-B analogue, and hence a consistent blank solution is not available. Indeed, any attempt to create a formulation without the fluorescent probe would not guarantee a proper baseline, as SP-B has a crucial role in determining the surfactant structure.

We considered that a solution to this issue can arise from the assumption that, under conditions where the fluorescent probe is completely quenched, the best baseline for each spectra data set is provided by the CHF5633 formulation itself. Hence, the information required to determine and subtract a dominating background signal that cannot be directly measured can actually be retrieved from the quenching experiment itself.

**Fig 3:**
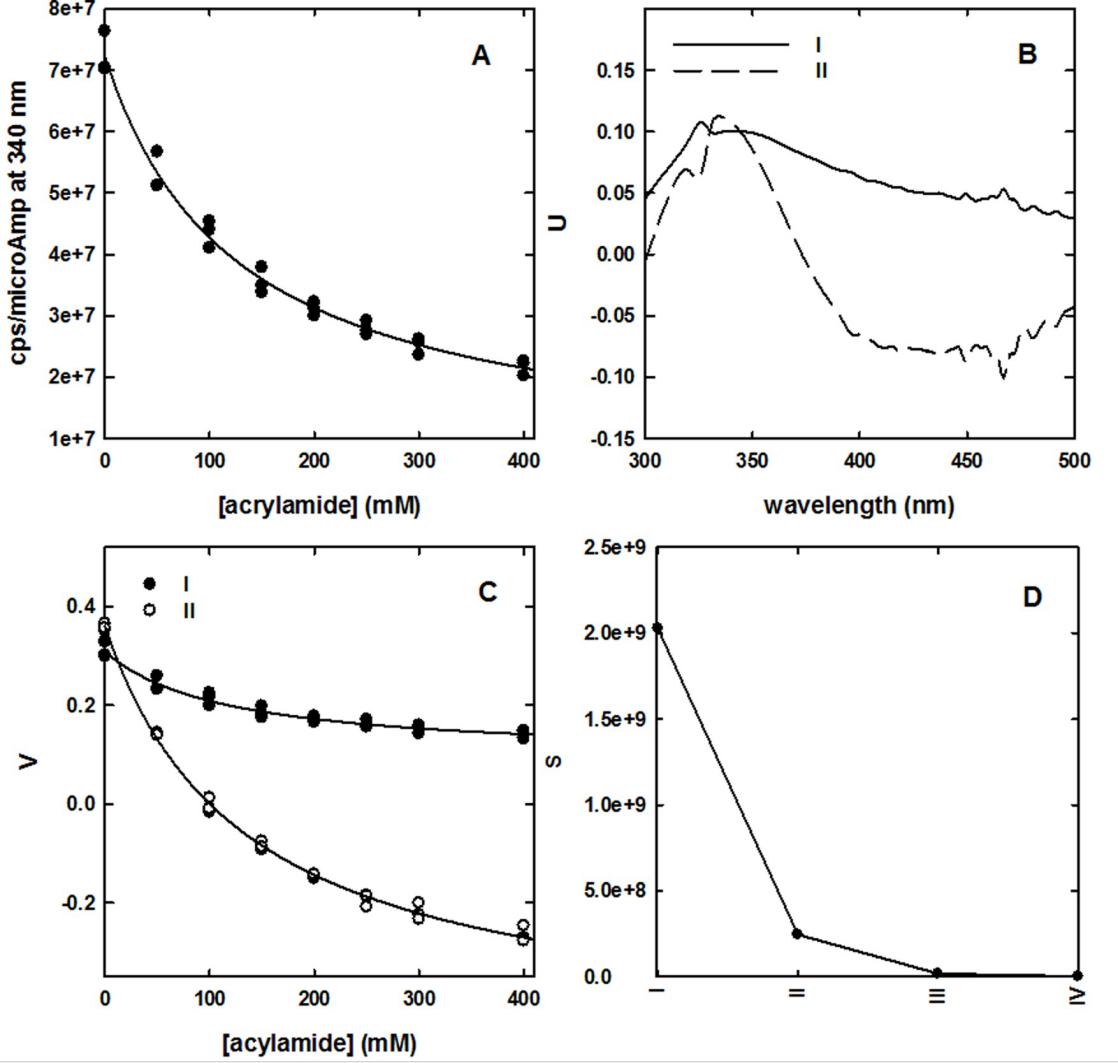
Baseline calculation by SVD analysis of the dependence of fluorescence emission on acrylamide concentration. A: Dependence on acrylamide concentration of fluorescence emission at 340 nm, upon excitation at 280 nm, at 20 ± 0.5°C. The solid line represents a hyperbolic fit of the experimental points (triplicates). B: First two principal spectral components retrieved by SVD analysis (U matrix). C: Dependence on acrylamide concentration of the relative contribution (V matrix) of the first two principal spectral components. D: four higher singular values S of spectral components retrieved by SVD analysis.

In Fig 3A, we plot the correlation between fluorescence emission at 340 nm and acrylamide concentration from the quenching experiment. It can be noticed that such dependence is well described by a hyperbolic function whose asymptote at infinite quencher concentration will correspond to the baseline contribution at this wavelength. No correction was applied for inner filter effects induced by acrylamide, that in our experimental settings has an absorbance well below 0.1 OD even at the higher concentration exploited. The good agreement of the experimental points (triplicate spectral acquisitions for each quencher concentration) with a simple hyperbolic decay suggests that the single Trp residue present in the formulation represents a homogeneous population in terms of accessibility to acrylamide. The reconstruction of the baseline spectrum by hyperbolic fitting (or by extrapolating y intercept on the double reciprocal plot) for each wavelength, even though possible, does not globally consider the spectral evolution at increasing quencher concentration. For this reason, we applied a singular value decomposition (SVD) analysis to the data matrix from the quenching experiments. This technique has wide applications in noise reduction in spectroscopic data matrices [40], and as a tool to extrapolate the spectra of intermediate species in kinetic series [37, 38]. The SVD analysis allowed to identify principal spectral components (U matrix), their relative contribution as a function of quencher concentration (V matrix) and their corresponding singular values (S matrix) (Fig 3B, 3C and 3D, respectively). We found that, based on singular values S, only the first two components are meaningfully emerging from noise, random components (Fig 3D). The dependence on acrylamide concentration of the V coefficients for the first two components was fitted to a hyperbolic model, and the asymptotic values were used to reconstruct the baseline by multiplying these extrapolated values for their corresponding U components and S values. The resulting spectrum, representing the baseline, is reported in Fig 2B (dashed line). Fig 2C shows a series of emission spectra in the absence and in the presence of different acrylamide concentrations where the generated baseline was subtracted. Scattered peaks present in the raw fluorescence data (Fig 2B) were almost completely removed.

Upon the subtraction of the baseline, the Trp emission peak resulted to be centered at 336 nm, independent of the concentration of acrylamide. The absence of peak shifting further suggests that SP-B analogue Trp residues are homogeneously distributed in terms of local microenvironment and acrylamide accessibility [30, 32, 33]. The maximum emission wavelength of 336 nm represents an intermediate value between those experimentally observed for Trp residues deeply buried in the hydrophobic protein core or highly exposed to aqueous solvents, ranging from ca. 310 to ca. 350 nm, respectively [29, 30, 32, 33, 41, 42]. The emission of Trp in SP-B analogue is almost identical to that of 335 nm observed for isolated N-term sequence of SP-B peptide (comprising helix 1 and 2) in the presence of 40 μM lipids (DPPC/PG) [31]. The same peptide exhibited emission at 347 nm in HEPES buffer at pH 7, very similar to Trp in aqueous solution [29, 31]. Other studies reported for full length SP-B, in organic solvent, emission at 330 nm, while SP-B in lysophosphatidyl choline micelles emits at 333 nm [43].

The SP-B analogue of CHF5633 lacks the first 7 aminoacids with respect to full length, wt SP-B. The 7 residues at the N-terminus constitute the membrane insertion sequence, which is reported to cause a dramatic increase in the acyl chain disorder in the center of the bilayer [44]. The Trp residue of SP-B analogue of CHF5633 reports the same polarity as Trp of peptides presenting the 7 amino acids insertion sequence, suggesting an unaltered location and a maintained capacity of anchoring on the membrane surface.

### Quenching of fluorescence emission by acrylamide: Accessibility of SP-B analogue tryptophan residue

**Fig 4:**
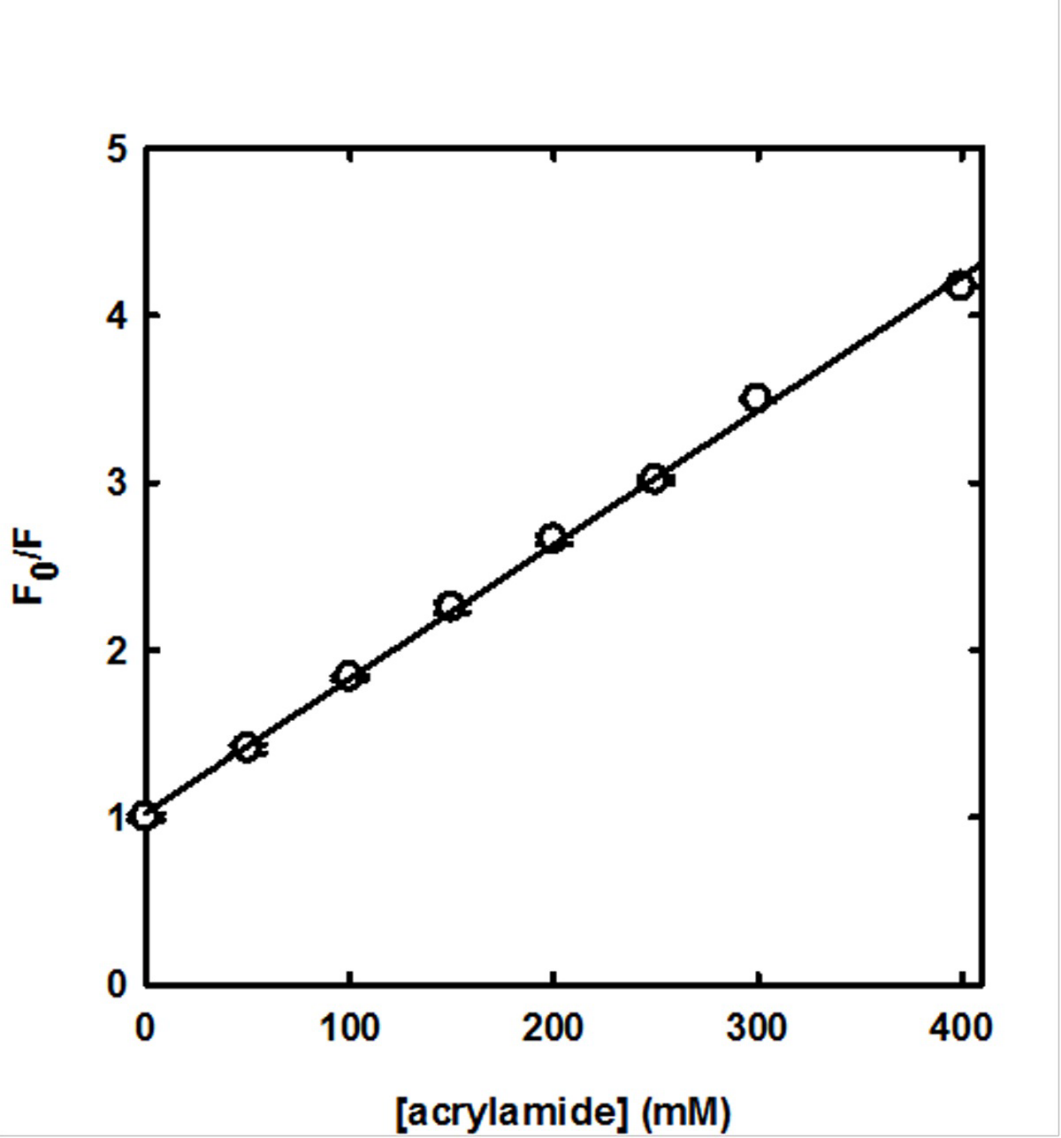
Quenching by acrylamide of SP-B analogue tryptophan fluorescence emission in synthetic surfactant. The ratio of fluorescence emission in the absence (F_0_) and in the presence (F) of acrylamide is plotted as a function of quencher concentration (λ_ex_ = 280 nm, λ_em_ = 340 nm, 20 ± 0.5°C). Experiments were carried out in triplicate and are reported as average ± SE. The solid line represents the fit to a linear regression, yielding R^2^ > 0.996 and a Stern-Volmer coefficient K_SV_ = 7.76 ± 0.14 M^-1^.

Quenching of fluorescence emission reports on the solvent accessibility of the fluorophore, thus representing a sensitive conformational probe of the protein region surrounding the chromophoric group [33]. Fluorescence quenching experiments of SP-B analogue in synthetic surfactant are described by the Stern-Volmer plot reported in Fig 4, where the ratio between the fluorescence emission in the absence (F_0_) and in the presence (F) of acrylamide is shown as a function of the quencher concentration.

No apparent upward or downward curvature, commonly related to heterogeneities in the quencher concentration due to partitioning or to the presence of different populations of fluorophores with distinct accessibilities [45], respectively, could be noticed. A linear relationship in the Stern-Volmer plot can be fitted to the equation:

$$\frac{F_{0}}{F}=1+K_{SV}\cdot \left[Q\right]$$

where K_SV_, the Stern-Volmer constant, is a measure of the accessibility of the fluorophore to the quencher. Fitting of the data reported in Fig 4 to a linear regression yields a R^2^ over 0.996, with a K_SV_ of 7.76 ± 0.14 M^-1^. The observed linear behavior indicates a homogeneous population of fluorophores in terms of quencher accessibility, and therefore, most likely, also in terms of protein conformation in the Trp microenvironment. As a control,the same experiment was carried out at 37°C, a physiologically relevant temperature where the lipid component is supposed to completely populate the fluid phase. Also at this temperature no upward or downward curvature was observed (data not shown), indicating that homogeneity of the spectral probe is maintained, independent of the prevailing phase experienced by membrane phospholipids. and Fitting to the Stern-Volmer equation yielded a K_SV_ of 11.30 ± 0.13 M^-1^.

Previous reports of fluorescence quenching studies carried out on micellar systems indicated significant deviations from linearity for saturation of the micelles at high concentration of quencher (iodide or H_2_O_2_) or quencher partitioning in the region of space containing the fluorophore [46]. The linear behavior observed for CHF5633 Trp quenching by acrylamide indicates that none of these two process occurred, in agreement with a scenario where, although SP-B analogue is anchored to the multilamellar vesicles of the surfactant, the high concentration of the vesicles does not allow quencher saturation (the phospholipid concentration is about 100 mM, to be compared with the 1 mM micelles concentration observed in the work of Cramb and co-workers [46]).

### Bimolecular quenching constant analysis: Tryptophan accessibility and SP-B analogue lateral diffusion within phospholipid bilayer

The linear behavior of tryptophan fluorescence quenching by acrylamide reported in Fig 4 suggests that we are observing a purely collisional (or dynamic) quenching, hence the slope K_SV_ corresponds to the fluorophore lifetime τ_0_ multiplied by the bimolecular quenching constant k_q_; the latter is correlated to k_0_ (diffusion controlled bimolecular rate constant) through the quenching efficiency f_q_ [29], *i*.*e*. the probability of a collisional encounter to be effective in quenching:

$$K_{SV}=\tau_{0}\cdot k_{q}=\tau_{0}\cdot f_{q}\cdot k_{0}$$

Tryptophan fluorescence in proteins is usually characterized by structured emission decays, with mean values that can range from less than 2 to more than 5 ns [47]. If we assume τ_0_ to be 2.7 ns, close to the average value for mean lifetime that can be calculated from a dataset of 10 different proteins, or that of Trp free in solution [47, 48], and K_SV_ = 7.76 (from our data, Fig 4) the calculated k_q_ is 2.9·10^9^ M^-1^ s^-1^. k_0_ can be obtained using Smoluchowski equation [49]:

$$k_{0}=\frac{4\cdot \pi \cdot N_{A}}{1000}\left(R_{f}+R_{q}\right)\left(D_{f}+D_{q}\right)$$

where N_A_ is the Avogadro number (6.022·10^23^), R_f_ and R_q_ the molecular radii of the fluorophore and the quencher, respectively, and D_f_ and D_q_ the diffusion coefficient of the fluorophore and the quencher, respectively.

R_f_ and R_q_ were assessed by determining the molecular volume of Trp and acrylamide using the ChemSketch software and calculating the radii of the equivalent spheres (3.90∙10^−10^ m and 3.08∙10^−10^ m, respectively).

The diffusion coefficient for acrylamide (D_q_) was calculated using the Stokes-Einstein equation:

$$D_{q}=\;\frac{k_{B}T}{6\pi \eta r}$$

where k_B_ is the Boltzman constant (1.38·10^−23^ J·K^-1^), r is the molecular radius, T is the temperature and η the viscosity. Considering the low solubility of acrylamide in apolar solvents, we assumed its full partitioning in water, and hence we applied water viscosity (~1 cPoise, 1 mPl). The calculated diffusion coefficient for acrylamide, D_q_, was 7·10^−6^ cm^2^·s^-1^.

The calculation of the diffusion coefficient for Trp, D_f_, required to take in consideration the fact that SP-B analogue is inserted/anchored to the lipid vesicles of the formulation, and consequently we could not simply consider random diffusion of the fluorophore in the medium. Indeed, the diffusion will depend also on the anisotropic nature of the medium in which the fluorophore, which is part of a peptide, is inserted (two dimensions are the phospholipid membrane surface and the third dimension is water surrounding vesicles). In membranes, the translational diffusion is much faster (about four times) than the rotational mobility [50]. Moreover, SP-B analogue has a marked partitioning of hydrophobic residues to one face and positively charged residues to the opposite face, making interaction with phospholipids constrained from the orientational point of view [15]. Hence, we did only consider lateral diffusion, applying the Saffman-Delbrück equation [50] for calculating the diffusion coefficient of Trp:

$$D_{f}=\;\frac{k_{B}T}{4\pi \eta h}\left(ln\frac{\eta h}{\eta^{\prime }r}-\gamma \right)$$

where η e η’ are the viscosities of the phospholipid bilayer (200 cPoise) [51] and of the liquid in which vesicles are dispersed (water, 1 cPoise), respectively, h is the membrane thickness (5 nm for a double layer), r the molecular radius and γ the Eulerian constant (0.5722). Since Trp is part of SP-B analogue peptide, and the lateral diffusion involves the whole peptide rather than the single aminoacid, we applied as a molecular radius that corresponding to the whole SP-B peptide (1 nm, extrapolated from [52]) and obtained a calculated diffusion coefficient (D_f_) of 2·10^−8^ cm^2^·s^-1^. The calculated radii and diffusion coefficients were then used for calculating k_0_ from the Smoluchowski equation, resulting to be 3.7·10^9^ M^-1^·s^-1^. The calculated k_0_ (diffusion controlled bimolecular rate constant) is very similar to the bimolecular quenching constant k_q_, hence the quenching efficiency f_q_ is near to unity (0.78), indicating that the tryptophan residue of SP-B analogue in synthetic surfactant is highly accessible to the acrylamide quencher and therefore, likely, significantly exposed to the aqueous phase of the formulation [32]. The quenching efficiency f_q_ was also calculated for acrylamide quenching experiments carried out at 37°C, for which a higher K_SV_ was observed (11.30 ± 0.13 M^-1^). Upon calculating k_0_ at a different temperature (viscosities of water and phospholipid bilayer correspond to 0.7 and 110 cPoise, respectively), the resulting f_q_ (0.75) resulted to be almost identical to that obtained at 20°C.

Considering the main partitioning of acrylamide into the polar phase, the measured quenching effect will be mostly affected by the bimolecular quenching constant of Trp side chains highly or fully accessible to the aqueous phase, suggesting that SP-B analogue is highly and homogeneously surface-exposed to the water in both gel and liquid phases.

To gain further insight on the accessibility of Trp residues of SP-B analogue in CHF533 synthetic surfactant, we took advantage of a modified version of the Stern-Volmer equation previously used by Hare and co-workers to determine the fraction of accessible or inaccessible Trp residues in the case of liposomes-inserted proteins [53]:

$$\frac{F_{0}}{F_{0}-F}=\frac{1}{f_{a}\cdot K_{a}\cdot \left[Q\right]}+\frac{1}{f_{a}}$$

where f_a_ is the fraction of accessible fluorophores and k_a_ the quenching constant.

We fitted to this modified Stern-Volmer equation the acrylamide quenching data reported in Fig 4. The fit yielded a f_a_ of 1.02 ± 0.01, supporting the view of a homogeneous population of Trp fully accessible to the quencher, even though belonging to a peptide anchored to the lipid bilayer.

These data confirm that the SP-B analogue within the synthetic surfactant adopts a surface localization on the lipid bilayer, as already demonstrated for SP-B full-length protein, for which FRET studies were consistent with a superficial orientation, with Trp always adopting a surface localization in the membrane [14]. In particular, a peptide orientation parallel to the membrane surface, establishing hydrophobic interactions between the amphipathic helical segments and the membrane surface, has been hypothesized [2, 43].

The high quencher accessibility of Trp might apparently be inconsistent with the observed maximum emission wavelength, which is suggestive of an only partially polar microenvironment ([30] and references therein). Indeed, the Trp emission peak at 336 nm is very similar to that of Staphylococcus nuclease [32, 33]. The latter, however, yielded a markedly lower K_SV_ (4.5 M^-1^). On the other hand, the k_q_ measured for acrylamide quenching of ACTH Trp was 4.2·10^9^ M^-1^·s^-1^ [32, 33], close to what we observe for the quenching of SP-B analogue within the formulation, and to the value expected for a quenching exclusively controlled by diffusion (k_q_ = 1·10^10^ M^-1^·s^-1^) [29].

**Fig 5:**
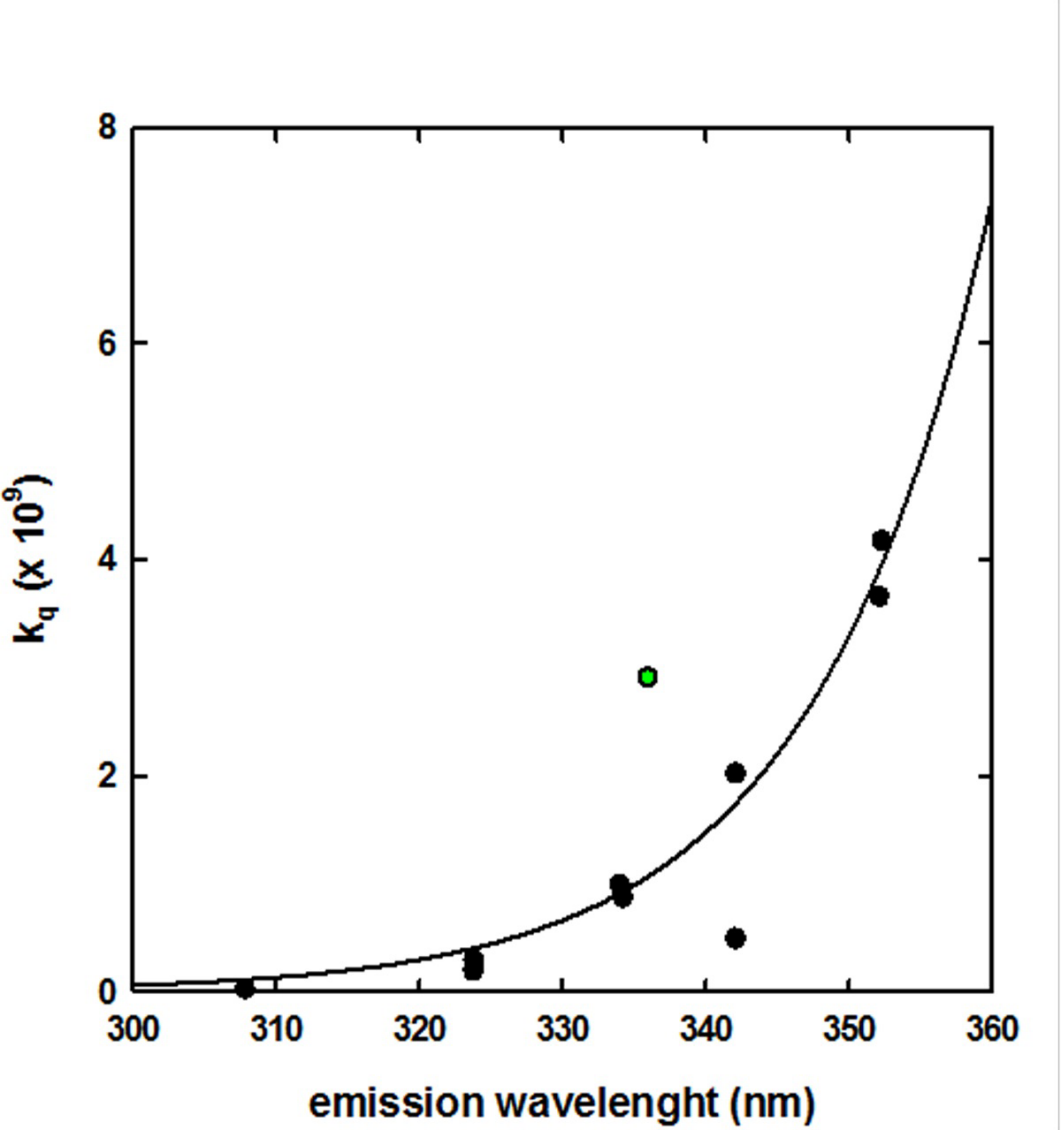
Correlation between bimolecular quenching constant k_q_ and Trp emission wavelength of a representative set of proteins: Black circles, data from Eftink and Ghiron (1976).

Eftink and Ghiron reported a correlation between k_q_ and emission peak wavelength for a series of proteins [33]. When we tried to overlap to their plot the data that we obtained for SP-B analogue in synthetic surfactant, we found the latter to represent a significant outlier (Fig 5).

This observation is consistent with the other results reported in the present study, which suggest that the Trp residue of CHF5633 SP-B analogue is highly exposed, and therefore likely does not take part to the inter-helix interaction, but is rather possibly involved in anchoring the peptide at the lipid-water interface. As a matter of fact, the apparent polarity of its microenvironment is significantly lower than that of a purely aqueous phase and in agreement with a localization of Trp at the level of head group of phospholipids, as often observed for membrane peptides and proteins [29]. An additional factor that could help to reconcile high Trp accessibility and intermediate apparent polarity resides in the highly lipophilic nature of SP-B sequence, given the known effect of sequence on Trp emission wavelength in proteins [30].

### Quenching of fluorescence emission by 10-doxyl nonadecane: Static quenching demonstrates stable interaction of SP-B analogue with phospholipid vesicles

**Fig 6:**
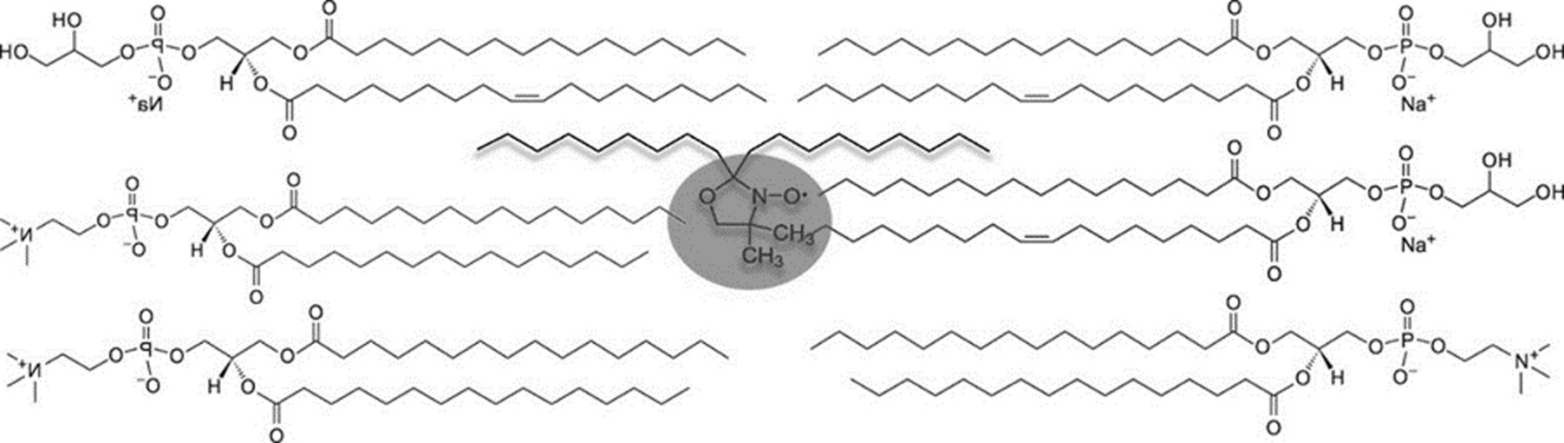
10-doxyl nonadecane intercalated within a phospholipid bilayer. The doxyl group is shadowed in gray.

To better evaluate the localization of SP-B analogue in the structured matrix of the synthetic surfactant formulation, we measured Trp fluorescence emission in the presence of the lipophilic quencher 10-doxyl nonadecane (10-DN). This molecule carries a doxyl group (quencher) placed in central position between two aliphatic chains that make 10-DN able to intercalate within double layer membranes (Fig 6).

In the CHF5633 synthetic surfactant, 10-DN is supposed to assume the same localization, with the quencher group at the center of the phospholipid bilayer of vesicles, so that its efficiency in quenching Trp fluorescence can be used as a probe of the localization of SP-B analogue with respect to the lipid bilayer, as similarly explored on full length SP-B [54]. This approach, with the combined use of the two quenchers acrylamide and 10-DN, has been previously proposed to determine the position within membranes of hydrophobic α-helices carrying Trp residues [34]. It has to be noted that, similarly to acrylamide, 10-DN quenching was shown to be relatively insensitive to variations of the lipid component (similar quenching efficiency for Trp at the center of a double layer of DEuPC, 1:1 DOPG/DOPC or DOPC).

**Fig 7:**
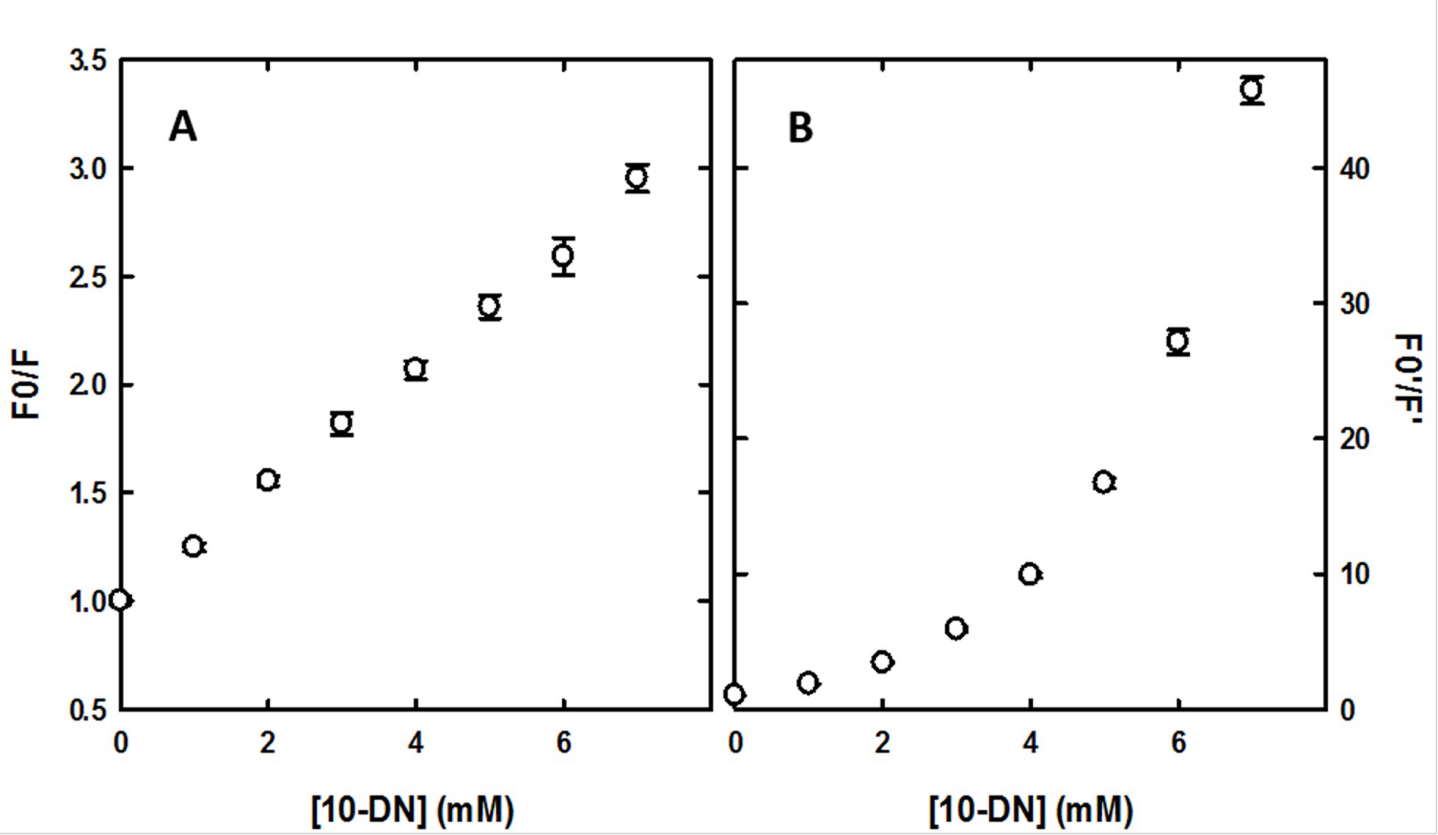
Stern-Volmer plots for quenching of SP-B analogue tryptophan fluorescence by 10-doxyl nonadecane. The ratio of fluorescence emission in the absence and in the presence of 10-DN is plotted as a function of quencher concentration (λ_ex_ = 280 nm, λe_m_ = 340 nm, 20 ± 0.5°C). Experiments were carried out in triplicate and are reported as average ± SE. A: raw data. B: data corrected for inner filter effect according to Leese and Wehry [55].

We carried out Trp fluorescence quenching experiments with 10-DN generating the baseline with the same procedure optimized for acrylamide. We did not observe any shift of the emission peak of Trp (336 nm) as a function of 10-DN concentration (data not shown), demonstrating that the addition of the lipophilic quencher did not alter the polarity explored by Trp and hence the localization of the SP-B analogue. Since SP-B is strictly interacting with the phospholipid bilayer, we may suppose that that the quencher does not alter its structure. However, differently from acrylamide, which absorption peak is centered at around 195 nm and absorbance at 280 nm can be ignored in our experimental conditions, it was necessary to consider 10-DN absorbance at the excitation wavelength (280 nm), since at high concentration the quencher absorbs part of the excitation light, causing an artifactual decrease of fluorescence emission (inner filter effect). Experimental fluorescence quenching data (Fig 7A) were corrected according to Leese and Wehry [55]. We considered that, in the case of an inner filter effect due to absorption of the quencher (Q) at the wavelength of excitation of the fluorophore (Trp), which is our case, and assuming monochromaticity of the incident radiation, in the absence of quencher (Q):

$$F_{0}^{'}=k\cdot F_{0}\cdot P^{0}\cdot \varepsilon_{F}\left[Trp\right]$$

and in presence of quencher:

$$F^{\prime }=k\cdot F\cdot P^{0}\cdot 10^{-\varepsilon_{Q}\cdot b_{eff}\cdot \left[Q\right]}\cdot \varepsilon_{F}\left[Trp\right]$$

where $F_{0}^{'}$ and $F^{'}$ is the fluorescence emission in the absence and presence of the quencher, corrected for the inner filter effect, F_0_ and F is the measured fluorescence emission, k is a constant that includes instrumental geometrical factors, P^0^ is the power of the incident light, ε_F_ and ε_Q_ are the molar extinction coefficients of the fluorophore and the quencher, respectively, and b_eff_ is the average optical pathway of the incident radiation, which is approximated, in the case of right-angle illumination, to half of the optical path of the cuvette (0.5 cm in our case).

Dividing the terms of the two equations above, we obtain:

$$\frac{F_{0}^{'}}{F^{\prime }}=\;\frac{F_{0}}{F}\cdot \frac{k\cdot P^{0}\cdot \varepsilon_{F}\left[F\right]}{k\cdot P^{0}\cdot 10^{-\varepsilon_{Q\cdot b_{eff}\cdot \left[Q\right]}}\cdot \varepsilon_{F}\left[F\right]}=\frac{F_{0}}{F}\cdot \frac{1}{10^{-\varepsilon_{Q}\cdot b_{eff}\cdot \left[Q\right]}}$$

from which we can calculate the corrected (F_0_'/F') quenching data, using ε_Q_ = 680 M^-1^ cm^-1^ and b_eff_ = 0.25 (half of the 0.5 cm cuvette optical path) (Fig 7B).

Following the correction for the inner filter effect, the Stern-Volmer plot showed an exponential trend, similar to that observed for other phospholipids labeled with the quencher nitroxide [56] and in the case of the same 10-DN [34]. This behavior reflects the fact that diffusion in the phospholipid bilayer is slow and the quenching of a molecule within the bilayer is dominated by the proximity between quencher and fluorophore rather than by collisional events. Hence, the observed quenching of SP-B analogue Trp fluorescence represents mainly a static quenching, consistent with a SP-B analogue peptide stably anchored to the surface of the lipid bilayer.

## Conclusions

Fluorescence quenching experiments were carried out on a complete pharmaceutical formulation of a synthetic lung surfactant, CHF5633 (Chiesi Farmaceutici, Italy). This work demonstrates the possibility to get structural information from steady state fluorescence spectroscopy of complex and turbid pharmaceutical preparations with no need for sample processing. Specifically, we show that fluorescence quenching can be applied on samples with inherently strong light scattering, such as highly concentrated multilamellar vesicles suspensions. The quenching experiment itself was used to obtain baselines for background subtraction, upon extrapolation at infinite quencher concentration and SVD analysis of spectroscopic signals. As for characterization of CHF5633 synthetic surfactant, acrylamide quenching highlighted that the Trp residue present in SP-B analogue sequence represents a conformationally homogeneous population of fluorophores, highly accessible to water-soluble quenchers. This suggests a SP-B analogue superficial localization on the membrane of phospholipid vesicles, similarly to what observed for full length SP-B in lung surfactant [14, 43]. Quenching experiments with the lipophilic quencher 10-DN confirmed the stable interaction of SP-B analogue with the multilamellar vesicles, again supporting the view that the mode of interaction of SP-B analogue with phospholipids is similar to that of the full-length protein in lung surfactant.
